# Isolation of a New *Acetobacter pasteurianus* Strain from Spontaneous Wine Fermentations and Evaluation of Its Bacterial Cellulose Production Capacity on Natural Agrifood Sidestreams

**DOI:** 10.3390/foods15010154

**Published:** 2026-01-03

**Authors:** Vasiliki Adamopoulou, Vasiliki Karakovouni, Panagiota Michalopoulou, Panagiota M. Kalligosfyri, Agapi Dima, Theano Petsi, Despina P. Kalogianni, Argyro Bekatorou

**Affiliations:** Department of Chemistry, University of Patras, 26504 Patras, Greece; adamopoul_v@upatras.gr (V.A.); pkalligosfyri@gmail.com (P.M.K.); agapidima@hotmail.com (A.D.); thpetsi@upatras.gr (T.P.); kalogian@upatras.gr (D.P.K.)

**Keywords:** bacterial cellulose, *Acetobacter pasteurianus*, Response Surface Methodology, substandard raisins, oranges, green tea, antioxidant activity

## Abstract

A bacterial cellulose (BC) producing bacterial species was isolated from spontaneous wine fermentations and identified as *Acetobacter pasteurianus* and assigned the strain designation ABBA. The strain had the ability to synthesize BC in orange juice, achieving a yield of 5.0 g/L. Further production optimization was studied using a non-fortified natural substrate composed of substandard raisin extracts, orange juice, and green tea extract. The Response Surface Methodology for the production design and optimization was applied, resulting in a significantly higher yield of up to 15.5 g/L. The porosity, crystallinity, and antioxidant activity of the produced BC films were affected by both the BC drying method and the substrate used. In the FT-IR spectra, characteristic peaks corresponding to citric acid, gallic acid, ascorbic acid and thiamine were observed, indicating their adsorption onto the BC matrix and explaining the increased antioxidant activity. *A. pasteurianus* ABBA is a promising new strain that can be used in the production of BC from agrifood sidestreams (substandard raisins; discarded oranges), contributing to their utilization and the production of value-added materials within a circular-economy framework.

## 1. Introduction

Bacterial cellulose (BC) is an ultra-fine nanofibrillar polymer noted for its distinctive physicochemical and mechanical characteristics, such as a high degree of polymerization and crystallinity, a large specific surface area, notable flexibility and tensile strength, and exceptional water-holding capacity [1,2,3]. Structurally, BC is composed of β(1→4)-linked glucan chains stabilized by hydrogen (H)-bonding, with the empirical formula (C_6_H_10_O_5_)_n_ [4]. Although most hydroxyl groups (–OH) participate in H-bonding, each cellulose chain retains an unmodified –OH at C4 and a free –OH at C1, allowing for further chemical functionalization [1].

Despite these advantageous properties, BC remains costly to produce. The price of the cultivation medium, often representing around 30% of total production expenses, together with the generally modest productivity of many BC-producing bacteria, limits large-scale manufacturing and broader commercial use [3,4,5]. As a result, interest has increased in identifying more economical fermentation substrates. Numerous alternative C-sources have been investigated, including food-industry residues and sidestreams and agricultural wastes, such as brewery by-products, molasses, straw, foliage, pulps, fruit peels, raisins, vegetables, tea waste, and dairy effluents [1,2,3]. These materials are promising feedstocks for BC production because they typically contain high levels of carbohydrates, proteins, and other nutrients essential for microbial growth [2].

Recently, Bekatorou et al. [6] utilized substandard raisin extracts (SRE), a sidestream from industrial raisins finishing, either alone or combined with whey and with or without the addition of N-sources, and achieved high BC yields in the plain SRE with yeast extract supplementation (18.3 g/L or 0.09 g/g of sugar). The SRE without any supplementation (20 g/L initial sugar, pH 6.8, and 7-day cultivation) yielded 8.7 g/L, which is also considered high compared to other reported yields from unoptimized substrates. Therefore, raisin extracts were considered a good substrate for further study. Andritsou et al. [7] reported successful BC production from citrus juices derived from low-grade fruits, attaining substantial productivity (6.1–6.7 g/L), while Adamopoulou et al. [3] employed a mixture of SRE, orange juice (OJ), and tea extract, supplemented with organic acids and vitamins. BC yields up to 19.4 g/L were achieved in SRE supplemented with ascorbic acid, thiamine, and gallic acid. Based on these studies, it is considered evident that natural substrates composed of raisin extracts, citrus juices, and other materials rich in vitamins and phenolic compounds are efficient for BC production. The use of diverse agro-industrial residues as C, N, and micronutrient sources not only enhances the sustainability of BC production but also helps mitigate environmental impacts [4].

Optimizing cultivation conditions is essential in microbial fermentation, as these parameters directly influence both the quantity and quality of the product [5]. Refining BC production variables has therefore become a key strategy for reducing operational costs and processing time while improving output, particularly in large-scale systems. Such optimization efforts typically involve assessing nutrient requirements, selecting robust and productive bacterial strains, adjusting fermentation conditions, and comparing yields across varying process configurations [8]. The choice of microorganism is especially critical for efficient cellulose biosynthesis. A range of bacterial genera, including *Azotobacter*, *Komagataeibacter* (formerly *Acetobacter* and *Gluconacetobacter*), *Pseudomonas*, *Salmonella*, and *Sarcina ventriculi*, as well as certain algal species, are capable of producing BC [3,8,9]. However, among Gram-negative BC-producing bacteria, *Komagataeibacter* species, belonging to the acetic acid bacteria (AAB), are widely regarded as the principal producers due to their consistently high BC yields [8].

*Acetobacter pasteurianus* is also capable of synthesizing BC. It is a Gram-negative member of the AAB group, known for its ability to incompletely oxidize alcohol and sugars. A common characteristic among most AAB species is their capacity to oxidize ethanol to acetic acid, except for *Asaia* spp., which do not produce acetic acid from ethanol, and *Saccharibacter* spp. and *Granulibacter* spp., which form negligible amounts [10]. The metabolism and growth of AAB are significantly enhanced in oxygen-rich environments, with optimal conditions typically at pH 5.5–6.3 and temperatures between 25 and 30 °C. Their preferred C-sources include glucose, mannitol, and ethanol. In nature, *A. pasteurianus* is found in sucrose-rich substrates such as fruit, flowers, and vegetables. It is also associated with wine spoilage during storage and maturation. It is naturally present on grape surfaces, from where it enters the winery environment and persists throughout alcoholic fermentation, though its population tends to decrease under high ethanol concentrations and limited oxygen conditions [11,12].

Conventional BC optimization often examines one variable at a time (e.g., C-source, N-source, pH, temperature), a slow and costly approach requiring many experiments. Statistical optimization methods address these drawbacks by assessing multiple interacting factors simultaneously, generating more reliable data, and pinpointing key variables with fewer experimental runs [13]. Among the statistical tools used for process optimization, Response Surface Methodology (RSM) is one of the most widely applied. It uses mathematical and statistical procedures to design experiments, generate predictive models, identify significant factors, analyze interactions, and determine optimal operating conditions [13]. RSM describes the relationships between independent variables and responses, visualized through response surface plots. Compared with conventional one-factor-at-a-time approaches, it requires fewer experiments, reducing both time and cost. Effective use of RSM begins with defining appropriate parameter ranges, commonly through designs such as the Central Composite Design (CCD). When statistically validated, RSM models can reliably predict responses from given variable values. In particular, the combination of RSM and CCD has yielded excellent results in optimizing BC production [3,6,8].

In the search for new strains capable of producing BC from a variety of sustainable resources, this study reports the isolation and identification of the new *A. pasteurianus* ABBA strain from wine fermentation environments. It also presents the optimization of BC production by this strain using RSM/CCD, employing various agrifood sidestreams (substandard raisins and oranges) as substrates with the aim of promoting their valorization and developing environmentally friendly bioprocesses for high-value biomaterials production.

## 2. Materials and Methods

### 2.1. Chemicals

Alpha-D-Glucose (Serva, Heidelberg, Germany). Yeast extract, 2,2-diphenyl-1-picrylhydrazyl radical (DPPH), Pimarisin, and Penicillin (Duchefa Biochemie, Haarlem, The Netherlands). Soy Peptone, and starch index (Carl Roth, Karlsruhe, Germany). Bacteriological agar, Gel Loading Dye Purple (6×) (Biolab, Zrt., Budapest, Hungary). Calcium carbonate, disodium hydrogen phosphate, and sodium carbonate (Penta, Prague, Czech Republic). Citric acid and acetic acid glacial (Chem-Lab, Zedelgem, Belgium). HPLC-grade tartaric acid, D-fructose, malic acid, bromocresol purple, Lugol’s solution, Fuchsin, Crystal Violet, and cedar oil (Merck, Darmstadt, Germany). Ethylenediaminetetraacetic acid (EDTA), tetramethyl-p-phenylenediamine, and Fehling’s reagent (Sigma Aldrich, Saint Louis, MO, USA). Ascorbic acid (Honeywell Fluka, Shrewsbury, UK). Standard iodine solution 0.5 mol/L (1 N) (CARLO ERBA, Reagents S.A.S, Val de Reuil, France). Folin–Ciocalteu (Fisher Chemical, Loughborough, Leicestershire, UK). NucleoSpin Tissue kit: DNA (Macherey-Nagel, Düren, Germany). DNA molecular marker ΦX174 DNA-HaeIII Digest 250 ng/μL (New England Biolabs, Ipswich, MA, USA), and Primers (Eurofins, Genomics, Ebersberg, Germany). Digestion Solution for COD Kit (Hach Lange GmbH, Düsseldorf, Germany). Platinum DNA Polymerase (Thermo Fisher Scientific, Waltham, MA, USA).

### 2.2. Raw Materials

Wines originating from the spontaneous fermentation of *Roditis* grape must from the Achaean vineyard in NW Peloponnese, Greece, were used.

Substandard raisins were sorted out during the processing of superior-quality Corinthian currants (*Vostitsa*), which were obtained from the Agricultural Cooperatives’ Union of Aeghion S.A. (Achaia, Peloponnese, Greece). The aqueous extract (SRE) was prepared by pasteurizing the substandard raisins in hot water, followed by maceration at 70 °C (1:1 *w*/*w*) until the extract reached approximately 80 g/L sugar (~4 °Be), as previously described [14].

Oranges of the *Navelina* variety were sourced from local producers (Ilia, Peloponnese, Greece). The juice (OJ) was extracted and clarified by cloth filtration. Commercial green tea leaves (Young Hyson, Ceylon green tea, Mlesna Hellas, Athens) were used to prepare the green tea extract (GTE). A concentration of 6 g/L tea leaves was infused in hot water (80 °C, 10 min), and the extract was then filtered to remove solid residues [3].

### 2.3. Isolation of AAB

For the isolation of AAB capable of producing BC, a sterile Glucose–Yeast extract medium (GY) was used, containing (g/L): 100 glucose, 5 yeast extract, and 3 peptone in deionized water (pH 6.56). Aliquots of 10 mL were dispensed into test tubes, and after cooling to 25 °C, 0.01% *w*/*v* pimaricin and 0.01% *w*/*v* penicillin were added aseptically to inhibit yeasts, molds, and Gram-positive bacteria, respectively [15]. Subsequently, 1 mL of spontaneously fermented wine was inoculated into each tube. The tubes were incubated at 30 °C for 4 days.

For cultivation of the isolated strain, sterile Hestrin–Schramm (HS) medium (g/L: 20 glucose, 5 yeast extract, 5 peptone, 2.7 Na_2_HPO_4_, 1.15 citric acid; pH 6) was used. After cooling to room temperature, the pellicle formed in the GY medium was aseptically transferred into HS medium, which was then incubated at 30 °C for 7 days. Streak plating was subsequently performed from the liquid culture onto sterile solid HS medium (supplemented with 20 g/L agar). Plates were incubated at 30 °C for 5–7 days [16]. Colonies obtained from HS agar were further streaked onto DSMZ Medium 105 (g/L: 100 glucose, 10 yeast extract, 20 CaCO_3_, 15 agar, pH 6) and incubated for 72 h [6].

### 2.4. Identification of AAB

#### 2.4.1. DNA Extraction

DNA isolation was performed using the NucleoSpin Tissue kit following the manufacturer’s instructions [17]. Initially, AAB was grown in HS medium. 200 μL of the bacterial culture was placed in a microcentrifuge tube. Then, 200 μL of Buffer T1 and 25 μL of Proteinase K were added, followed by 200 μL of Buffer B3. The mixture was vortexed thoroughly and incubated at 70 °C for 10–15 min. Subsequently, 210 μL of ethanol (96–100%) was added and mixed vigorously. The lysate was transferred to a NucleoSpin Tissue filter unit placed in a collection tube and centrifuged for 1 min at 11,000× *g*. The flow-through was discarded, and the filter was returned to the collection tube. Two washing steps followed: 500 μL of Buffer BW and 600 μL of Buffer B5, each followed by centrifugation for 1 min at 11,000× *g* to clean the membrane on which the DNA was bound. After washing, the filter was placed in a clean 1.5 mL microcentrifuge tube, and 50 μL of Buffer BE was added for elution. Following a 1-min incubation at room temperature, the sample was centrifuged for 1 min at 11,000× *g* to recover the purified DNA in the eluate.

DNA concentration (ng/μL) and purity were determined using an Implen NanoPhotometer P-Class version 2.1 (Implen GmbH, Munich, Germany). First, 2 μL of Buffer BE was placed in the measurement chamber (Lid 10), followed by 2 μL of the DNA sample. Absorbance at 260 nm and 280 nm was measured, and the DNA concentration and A260/280 purity ratio were calculated.

#### 2.4.2. Polymerase Chain Reaction (PCR)

The isolated DNA was amplified by PCR using oligonucleotide primers for 16S rDNA amplification, which targeted conserved regions at the 5′ (16Sd: 5′-GCTGGCGGCATGCTTAACACAT-3′) and 3′ ends (16Sr: 5′-GGAGGTGATCCAGCCGCAGGT-3′) [18].

Sample preparation was carried out in a Telstar Mini-V/PCR vertical laminar flow hood. The final PCR volume was 25 μL, consisting of 9.5 μL double-deionized water, 12.5 μL 2× Kapa 2G Fast Master Mix, 1 μL forward primer (10 pmol/μL), 1 μL reverse primer (10 pmol/μL), and 1 μL target DNA. A negative control was also prepared by replacing the DNA with the same volume of deionized water to check for potential contamination. All samples were mixed thoroughly and placed in a PCR thermal cycler to initiate the amplification program. The PCR conditions were: initial denaturation step for 3 min at 95 °C, followed by 35 cycles of 1 min at 95 °C, 45 s at 52 °C, and 1 min at 72 °C. A final extension step at 72 °C for 7 min was performed.

#### 2.4.3. Agarose Gel Electrophoresis (AGE)

PCR products were visualized and quantified by agarose gel electrophoresis (AGE) using TAE buffer and ethidium bromide (EtBr) according to standard protocols [19]. For the preparation of the electrophoresis buffer, 20 mL of 50×TAE (Tris-acetate-EDTA) was added to a 1000 mL volumetric flask and diluted with deionized water; then 50 μL of 10 mg/mL EtBr was added. To prepare the gel, 1 g of agarose was mixed with 40 mL of the electrophoresis buffer in a conical flask and microwave-heated for approximately 2 min. Meanwhile, the electrophoresis tray was assembled, and a comb was inserted to form the sample wells. After the agarose was fully dissolved, the solution was allowed to cool slightly and then poured into the tray. Once solidified (20 min), the comb was removed, and the gel was placed in the electrophoresis tank containing 1×TAE buffer.

For sample loading, 5 μL deionized water, 2 μL 30% glycerol, and 5 μL of PCR product were mixed in a microcentrifuge tube. For the DNA molecular marker, 8 μL deionized water, 2 μL 6× loading dye, and 2 μL of a 250 ng/μL marker solution were combined.

The samples were loaded into the wells, and electrophoresis was performed at 90 V for 60 min to obtain a clear separation of the PCR products. After electrophoresis, the gel was exposed to UV light to visualize the bands, which were photographed digitally. Quantification was carried out using the Gel Analyzer plugin of ImageJ version 1.54g (NIH, Bethesda, MD, USA) based on a calibration curve generated from DNA markers of known molecular weight and the optical density of the electrophoretic bands.

#### 2.4.4. DNA Sequencing

DNA sequencing was performed using the Sanger method. Results were identified using the BLAST Nucleotide (BLASTn) online platform [20].

### 2.5. Optimization of BC Production

The optimization of BC production by the isolated strain in mixed agrifood substrates was based on a previous study with some modifications [3]. Specifically, for the optimization setup, 100 mL of sterile OJ, SRE, and GTE mixtures (pH 3.2) at various proportions were added to conical flasks. The substrates were sterilized (120 °C, 1–1.5 atm, 15 min), and then 15 mL portions were transferred to sterile Petri dishes and inoculated with 1 mL of the isolated strain stock culture (0.05 g/mL cell mass) under aseptic conditions. The dishes were then incubated at 30 °C for 7 days. The resulting BC gels were drained, placed on pre-weighed filter papers, and dried at 70 °C overnight. They were subsequently weighed again to calculate the BC yields, expressed as grams of BC per liter of substrate.

To optimize BC production under static culture conditions, the RSM/CCD methodology was applied to correlate the BC yield (g/L) (output) with the levels of several independent (input) variables (X_n_) affecting it. The experimental design involved two factors at two levels (2^2^ full factorial design) with five replications at the central point.

A total of 13 trials were conducted, in which two independent variables were examined: the SRE (X_1_) and the GTE (X_2_) concentration. OJ was used as the main substrate for BC production. For each independent variable, three experimental levels were used with coded values: −1 corresponding to the lower value, 0 to the center point, and +1 to the upper value (Table S1). The combinations of the independent variables, from the experimental design, are presented in the Section 3. The experiments were repeated in triplicate.

The concentration ranges of SRE and GTE used in the RSM design were determined based on preliminary screening experiments. These experiments showed that higher concentrations of SRE inhibited microbial growth and completely suppressed BC production, while lower concentrations resulted in suboptimal BC yields. Likewise, for GTE, it was observed that at higher concentrations in the mixed substrate, BC did not develop, while at lower concentrations, the yield was not satisfactory. Therefore, intermediate concentration ranges were selected to ensure microbial growth and adequate BC production.

The second-degree polynomial equation, which describes the predicted response as a function of the 2 independent variables, has the general form of Equation (1):

(1) $$Y=\beta_{0}+\Sigma_{i}^{k}\beta_{i}X_{i}+\Sigma_{j}^{k}\beta_{j}X_{j}^{2}+\Sigma_{i<j}\beta_{ij}X_{i}X_{j}+\varepsilon$$

where *Y* is the predicted response (BC yield), *β*_0_ is the model constant, *β_i_* is the linear effect coefficient, *β_j_* is the quadratic effect coefficient, and *β_ij_* is the interaction coefficient. *X_i_*, *X_j_* are the coded variables under study, and *ε* is the error.

An ANOVA analysis was performed, and the coefficient of determination R^2^ and the F-test value were calculated. The experimental value was compared with the predicted BC yield value [3].

### 2.6. Analytical Methods

#### 2.6.1. Microbiological Analysis of the Isolated Strain

To observe the morphology of the bacteria under an optical microscope, a colony was transferred from the solid medium (HS with agar) onto a slide, and the preparation was heat-fixed. Staining was performed with crystal violet solution for 1 min. A small amount of cedar oil was placed on the slide, and the sample was examined using the immersion lens (×100).

For Gram staining, a colony was transferred and homogenized in a drop of deionized water on a slide. The preparation was heat-fixed, staining was performed with crystal violet solution for 1 min, the excess dye was rinsed off with water, and a few drops of Lugol’s iodine (I_2_/KI) solution were applied for 3 min. The preparation was then rinsed with ethanol (dropwise for 1–3 min) followed by water. Only Gram-positive cells retain the blue color. The preparation was then covered with fuchsin for 1–2 min and rinsed again with water. A drop of cedar oil was added, and the sample was examined under an optical microscope at ×100 magnification [10].

For the catalase test, a few drops of 10% H_2_O_2_ solution were placed in a sterile Petri dish. Under aseptic conditions, a portion of cells from the isolated strain was transferred with a loop and mixed with the H_2_O_2_ solution. The presence or absence of foaming was observed; foaming indicates a positive reaction [10].

For the oxidase test, filter paper was placed in a sterile Petri dish and moistened with 1–2 drops of tetramethyl-p-phenylenediamine reagent. Using a sterile toothpick and working under aseptic conditions, a small number of cells from the isolated bacterial strain were transferred onto the filter paper. The appearance of a purple color within 20–30 s indicates a positive oxidase reaction. The test must be read no later than 60 s, as longer exposure may produce a false positive result due to auto-oxidation of the reagent in the presence of O_2_ [10].

For the acetic acid production test, a sterile solid medium (GY–CaCO_3_; GYC) was used, containing (g/L): 100 glucose, 5 yeast extract, 1.2 CaCO_3_, 3 peptone, and 20 agar in deionized water. The GYC medium was cooled to 40 °C and poured into Petri dishes. A small quantity of cells was plated, and the dishes were incubated at 30 °C for 5–7 days. Acetic acid produced by the culture reacts with CaCO_3_, causing a clear zone or discoloration of the medium around the colony [21].

For the peroxidation test, a sterile Carr medium was used, containing (g/L): 30 yeast extract, 0.022 bromocresol purple, 20 agar, and 2% *v*/*v* ethanol (95% *v*/*v*) in deionized water. The Carr medium was cooled to 40 °C and poured into Petri dishes. A small quantity of cells was plated, and the dishes were incubated at 30 °C for 14 days. A color change in the medium from purple to yellow, maintained throughout the 14 days, indicates that the isolated strain belongs to the genus *Gluconobacter*. In contrast, a change from yellow back to purple after 14 days indicates that the strain belongs to the genus *Acetobacter* or *Gluconacetobacter* [21].

For the ethanol oxidation test (oxidation of ethanol to acetic acid), a sterile synthetic solid medium (Ethanol–Yeast extract–CaCO_3_; EYC) was used, containing (g/L): 10 yeast extract, 20 CaCO_3_, 10 agar, and 3% *v*/*v* ethanol (95% *v*/*v*) in deionized water. The EYC medium was cooled to 40 °C and poured into Petri dishes. A small number of cells were plated, and the dishes were incubated at 30 °C for 5–7 days. Acetic acid produced by the oxidation of ethanol reacts with CaCO_3_, resulting in a clear zone or discoloration around the colony [21].

For the dihydroxyacetone (DHA) production test, a sterile synthetic medium (YG medium) was used, containing (g/L): 30 glycerol, 5 yeast extract, and 10 peptone in deionized water. The YG medium was cooled to room temperature. Three test tubes were prepared: (1) blank 1 (medium only), (2) blank 2 (medium plus Fehling’s reagent), and (3) sample (medium, Fehling’s reagent, and the microorganism). The tubes were incubated in a heating chamber at 30 °C for 4 days. On the 4th day, Fehling’s reagent was added to the sample tube, and the appearance of an orange color indicated a positive reaction [22].

For the BC production test, the liquid HS medium and the solid DSMZ Medium 105 were used. Under aseptic conditions, the isolated strain was transferred to Petri dishes containing the sterile medium using an inoculation loop. The plates were incubated at 30 °C for 7–10 days.

All experimental procedures were performed with 5 repetitions.

#### 2.6.2. Determination of Sugars and Organic Acids

For sugars and organic acids analysis, a LC-2000 Series HPLC system was used (JASCO Inc., Tokyo, Japan) equipped with Rezex ROA-Organic Acid H+ (8%) LC column (300 × 7.8 mm i.d., 8 μm p.s.; Phenomenex, Torrance, CA, USA), CO-2060 oven (33 °C), PU-2089 pump, MD-2018 photodiode array detector (210 nm) (for organic acids) and RI-4030 detector (for sugars), ChromNav 2.0 software, and mobile phase 0.005 M H_2_SO_4_ (0.5 mL/min) [3]. All samples were filtered through 0.22 µm syringe filters. Concentrations for all analytes were determined from standard curves constructed using standard solutions of 0.05, 0.1, 0.5, 1.0, 1.5, and 5% *w*/*v*.

#### 2.6.3. Determination of Vitamin C and Total Phenolic Content (TPC)

For vitamin C determination, 20 mL of each sample, 150 mL of distilled water, and 1 mL of starch indicator (1% *w*/*v*) were added to a 250 mL conical flask. The samples were titrated with a standard 0.005 M I_2_ solution. The endpoint was identified as the first permanent appearance of a dark blue color due to the starch–iodine complex [3].

The Folin–Ciocalteu method was used to determine the TPC. A 0.1 mL aliquot of sample, 5 mL of water, and 1 mL of Folin–Ciocalteu reagent were added to a 10 mL volumetric flask and kept in the dark for 30 min. Then, 1 mL of 7.5% *w*/*v* Na_2_CO_3_ solution was added, the volume was adjusted with water, and the flask was again placed in the dark for 30 min. Absorbance was measured at 725 nm using a JASCO V-630 UV–Vis spectrophotometer (JASCO Inc., Easton, MD, USA) against a blank. The results were expressed as gallic acid equivalents (mg GAE/L) [23].

#### 2.6.4. Determination of Chemical Oxygen Demand (COD)

The organic load of the substrate wastewaters after BC production was estimated by the determination of their COD at a 1:10 dilution. The analysis was carried out by a photometric test (USEPA Reactor Digestion Method, Method 8000; Hach Company/Hach Lange GmbH, Düsseldorf, Germany), using the Hach COD digestion reagent vials (range 0–15,000 ppm). In total, 2 mL of sample was added at a 45° angle to each vial. Deionized water was used instead of the sample for the blank assay. Digestion took place at 150 °C for 2 h in a Hach DRB-200 device, and after cooling, the Abs was measured on a Hach DR/2400 spectrophotometer (Hach Co., Loveland, CO, USA) [14].

#### 2.6.5. Physicochemical Characteristics and Antioxidant Activity

The physicochemical characteristics of the produced BC films were determined as previously described [3,6]. In brief, porosity characteristics (0.1–0.2 g sample; degassed under N_2_ at 95 °C for 120 min) were determined by N_2_ adsorption/desorption at 77 K over a wide range of relative pressures using a TriStar 3000 porosimeter (Micromeritics Instrument Corp., Norcross, GA, USA).

For Fourier-transform infrared (FT-IR) analysis, 2 mg of the sample was mixed with 200 mg KBr and pressed in a hydraulic press (8 tons) for 5 min. Spectra in the range 4000–500 cm^−1^ were recorded on a PerkinElmer spectrometer (Waltham, MA, USA) with a resolution of 4 cm^−1^. Each sample was scanned 10 times.

X-ray diffraction (XRD) patterns were obtained using a Bruker D8 Advance X-ray diffractometer (Bruker AXS, Billerica, MA, USA) with Ni-filtered CuKα radiation. The intensity of the diffracted radiation was measured in the range 5–60° (2*θ*) at a scan rate of 0.1°/min. The crystallinity index (CI) was calculated using the Segal equation.

The samples were coated with gold using a Balzers SCD 004 Sputter Coater (Oerlikon Balzers, Balzers, Liechtenstein) for 3 min, and studied on a JEOL JSM-5600LV Scanning Electron Microscope (SEM) (Jeol, Boston, MA, USA).

Finally, the BC films were also analyzed for antioxidant activity in both freeze-dried (FD) and oven-dried (OD) forms. OD was performed at 70 °C until constant weight, while FD was conducted by freezing at −44 °C and drying at −45 °C for 48 h at 5–15 mbar (FreeZone 4.5 System, Labconco, Kansas City, MO, USA). The antioxidant activity was determined by the DPPH radical scavenging method. A 50 mg BC film was mixed with 5 mL of 137.6 μM DPPH in methanol, and the absorbance (A) at 517 nm was measured before and after incubation for 30 min in the dark. The results were expressed as A% relative to a blank [3].

#### 2.6.6. Statistical Analysis

The significance of differences in the means of various data groups was checked by One-Way Analysis of variance (ANOVA), at the 0.05 level of significance, using the Microcal™ Origin^®^ software, v. 6.0 (Microcal Software, Inc., Northampton, MA, USA).

## 3. Results and Discussion

Several studies have explored sustainable BC production using low-cost substrates such as agrifood wastes and sidestreams and plant extracts. The present study contributes to this field in two novel ways: (i) by employing a newly isolated BC-producing strain obtained from wine fermentation environments, which is compatible with the fermentation of raisin extracts and is of relevance from a fundamental microbiological perspective; and (ii) by utilizing substandard raisins and citrus waste (discarded oranges) as substrates. This approach builds on previous work [3,6,14] and aims to valorize these agrifood sidestreams, reduce environmental impact, support the declining raisin sector, and enhance Mediterranean biodiversity.

### 3.1. Characteristics of the Isolated Strain

In Table 1, the microbiological characteristics of the newly isolated AAB strain are shown.

During its growth in liquid GY medium, the strain developed a gel-like membrane on the surface. Cells taken from the liquid culture were examined under an optical microscope, and their morphology was determined. The bacteria exhibited ellipsoidal (rod-shaped) morphology, with cells observed singly, in pairs, in chains, or in clusters. Gram staining showed that the strain is Gram-negative, as the cells appeared red.

Regarding the oxidase and catalase tests, the strain gave a negative oxidase reaction, as no purple coloration was observed, and a positive catalase reaction, as foaming occurred upon contact with H_2_O_2_ [10]. To determine whether the strain produces acetic acid and therefore belongs to the AAB group, growth on GYC medium was assessed. After 5 days, small white colonies developed, surrounded by zones of discoloration caused by the reaction of acetic acid with CaCO_3_ [21].

To determine whether the isolated strain is capable of acetic acid peroxidation, the synthetic Carr medium was used. The strain gave a positive result, as the medium changed from purple to yellow (day 5) and returned to purple by day 8, indicating that the bacterium converted acetic acid to CO_2_ and H_2_O [21,24].

Oxidation of ethanol to acetic acid was also examined on EYC medium. After 5 days, white colonies were observed, surrounded by CaCO_3_ discoloration zones, confirming the production of acetic acid.

After the formation of a gel in GY medium, it was also necessary to determine whether the strain could grow in HS medium and in the solid DSMZ Medium 105. Growth was confirmed, as colonies were formed in the solid media. In the BC production test, the strain grew in liquid HS medium, forming a membrane at the air–liquid interface.

Finally, in the DHA (ketogenesis) test, the strain gave a negative result, as no orange coloration developed upon addition of Fehling’s reagent after 4 days of growth [22].

These analyses were conducted to determine whether the newly isolated strain belongs to the AAB group and to identify its genus. Based on the results, the strain belongs to the genus *Acetobacter* or *Gluconacetobacter* and is capable of producing BC. These genera are characterized by rod-shaped, Gram-negative, catalase-positive, oxidase-negative, ethanol-oxidizing bacteria. They are also positive for acetic acid peroxidation, which distinguishes them from the genus *Gluconobacter*, which gives a negative result. Moreover, both *Acetobacter* and *Gluconacetobacter* include strains capable of producing BC [10,16,21,22].

### 3.2. Molecular Identification of the New Strain

Table 2 presents the concentration (ng/μL) and purity of the isolated DNA, which were determined by the absorbance (A) at 260 and 280 nm (wavelength absorbed by proteins), respectively.

When the A260/A280 ratio is between 1.8 and 2.0, the isolated DNA is considered pure [25]. The concentration of the isolated DNA was 44.5 ng/μL, with a purity ratio of 1.880. According to Boesenberg-Smith et al. [25], this value falls within the desired range, indicating that the isolated DNA is free of protein impurities. Its concentration is also sufficient, as it is close to the recommended value of 50 ng/μL.

After confirming the concentration and purity of the DNA isolated from the new strain, species-level identification was performed using 16S rRNA gene sequencing [26]. PCR amplification and electrophoresis of the PCR products were performed, and the PCR product appeared at 1353 bp (Figure S1).

DNA sequencing using the Sanger method was followed (Table S2). To identify the new strain, the BLAST Nucleotide tool was used, and the results showed that the strain belongs to the family *Acetobacteriaceae* and the species *Acetobacter pasteurianus*. The newly isolated strain is hereinafter referred to as *A. pasteurianus* ABBA.

### 3.3. Optimization of BC Production and Model Validation

The OJ in the optimization experiments for BC production remained constant as the basic substrate for *A. pasteurianus* ABBA, and the independent variables were the SRE and the GTE. After collecting the experimental results, a mathematical model was developed to describe the relationship between the dependent variable (BC yield) and the two independent variables. The BC yield is reported as volumetric productivity (g/L), as this is the standard metric in the majority of available literature and enables direct comparison across strains, substrates, and cultivation conditions. Since BC production is influenced by the overall medium composition, including the combination of nutrients and growth factors present, rather than by the C-source alone, this expression more accurately reflects process performance. Table 3 presents the means and standard deviations of the three replicates, as well as the predicted values obtained from the model.

It appears that there is good agreement between the experimental and predicted values, indicating that the conducted experiments align well with the model and demonstrate satisfactory reliability. The results were then mathematically processed to determine the optimal combination of independent variable values influencing the dependent variable. Linear regression was applied to the data, and a second-degree multiple linear regression equation was derived to describe the relationship between the dependent variable and the independent variables:

BC (g/L) = 6.341 + 1.152X_1_ + 0.154X_2_ − 0.005X_1_X_2_ − 0.050X_1_^2^ − 0.004X_2_^2^ (2)

The statistical significance of the model was determined using the F-test for ANOVA (Table S3). The regression was significant at the 5% significance level (*p* < 0.05), while the F-value of the model (3.35) indicates that it is statistically significant. The *p*-value (0.0073) is sufficiently small, further confirming the significance of the model. The coefficient of variation (CV) was 4.38%, which is considered satisfactory. The coefficients of determination, R^2^ and adjusted R^2^ (R_Adj_^2^), were 0.71 and 0.89, respectively.

**Figure 1:**
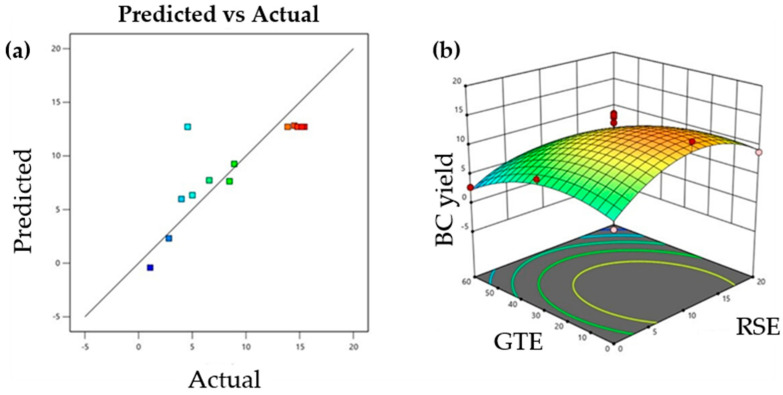
(**a**) Predicted values against experimental data of BC yield (g/L), according to the experimental design, and (**b**) 3D-imaging of the BC yield response surfaces at varying combinations and concentrations (g/L) of: SRE and GTE in OJ. Colors represent the predicted response values, with blue/green indicating lower values and yellow/red indicating higher response values.

Figure 1a shows the correlation between the model values and the experimental data. The model can be considered reliable for predicting BC production, as the data follow a normal distribution and the experimental values exhibit an almost linear correlation with the model’s predicted values. Figure 1b presents the three-dimensional response surface of BC production under static conditions as a function of the SRE and GTE content in the substrate. The maximum BC yield is observed at the central point, while both the highest and lowest concentrations of SRE and GTE lead to a decrease in BC yield.

The predicted BC yield from the mathematical model is 12.73 g/L. This value was verified experimentally by determining the BC yield based on the average of three replicates. The maximum experimental yield was found to be 15.44 ± 0.02 g/L, which is higher than the value predicted by the model, as the quadratic model provides a smoothed approximation based on all experimental data rather than fitting individual extreme values. Experimental variability and biological fluctuations may result in higher yields in specific runs. Nevertheless, the model (R^2^ = 0.71) effectively captures the overall trends and interactions among variables and remains suitable for optimization within the studied conditions, consistent with observations in our previous studies [3,6].

The optimized substrate composition was developed using SRE from substandard Corinthian currants, the same type (same grape variety and geographical origin) as reported in previous studies [3,6,14], but from a different vintage. Despite this variation and the fact that a different BC-producing AAB strain was used, the substrates consistently supported BC production, suggesting that the optimized formulation is reasonably robust to natural variability in the raw material. While a formal robustness study with multiple batches was not performed, these results indicate that the substrate composition can tolerate typical variations in raw material quality. The application of RSM, which evaluates system behavior across a defined experimental space, further supports the robustness of the optimized substrate composition by accounting for variability in substrate concentration and process conditions rather than relying on a single fixed formulation.

Generally, BC yields depend strongly on the substrate type, nutrient supplementation, incubation time, and the used strain. For example, *Komagataeibacter* spp. produce low, single-digit BC yields (g/L) on raw natural substrates, but higher yields (>10 g/L) are achievable when the same natural substrates and process conditions are optimized. Yields typically range from about 0.3 to 3 g/L in raw, unoptimized materials such as fruit juices or plant extracts. When agro-industrial wastes (e.g., molasses, fruit residues, etc.) are moderately optimized (e.g., by basic supplementation and improved culture conditions), yields commonly increase up to 10 g/L. Under highly optimized conditions (using selected high-producing strains, fortified waste streams, and controlled fermentation parameters), the reported BC yields may reach 10–20 g/L [1,2,3,4,5,6,7,8,9,12,13,14,27,28,29,30], as also reported in this study. The wide variation in BC yields reflects differences in strain performance, substrate composition, oxygen availability, and process conditions, including the fermentation duration. Therefore, agro-waste substrates such as molasses, fruit residues, and plant extracts, when optimized, can enable BC yields comparable to or better than standard HS glucose media.

Specifically, for *A. pasteurianus,* reported studies show that it generally produces moderate amounts of BC compared with top *Komagataeibacter* producers. Depending on the strain and substrate, yields typically fall between about 0.1 and 8 g/L under static cultivation. The higher yields (6–8 g/L) have been reported for selected strains grown on nutrient-rich media such as tomato juice or standard HS media, while lower yields (1–3 g/L) are more common when untreated agro-wastes are used. Overall, *A. pasteurianus* can synthesize BC, but its productivity is generally lower and more substrate-dependent than that of specialized *Komagataeibacter* strains [11,31,32,33,34].

The *A. pasteurianus* ABBA strain was stored in 25% (*v*/*v*) glycerol at −18 °C. The stability of its BC-producing ability was indirectly assessed through cultivation experiments performed at different time points using stored cultures, indicating that the strain retains its high BC-producing capacity after storage and recultivation. These observations support the suitability of the strain for further scale-up and industrial applications. Moreover, given that *Acetobacter* species are obligate aerobes, future work could focus on evaluating the feasibility of scaling up BC production from static cultures to agitated and/or aerated bioreactor systems. Such studies can investigate the effects of enhanced oxygen transfer and hydrodynamic conditions on BC yield and structure, as well as the potential need to adjust the optimized substrate concentrations to accommodate differences in mass transfer and shear stress under dynamic cultivation conditions.

Finally, among the natural substrates tested, tea represents a low-cost option as an N-source. It contains polyphenols, proteins, and amino acids, components known to supply nitrogen, minerals, and other essential nutrients to the growth medium of AAB [8,35,36,37,38]. It has been previously reported as an effective and low-cost N-source, enhancing BC yield and influencing material properties when used as a culture medium component [35,36,37,38]. This effect is attributed to the presence of organic nitrogen and minerals that support bacterial growth and metabolic activity, thereby indirectly increasing the availability of precursors required for BC biosynthesis [38]. In addition, tea polyphenols have been shown to affect microbial physiology and redox balance, which may modulate regulatory pathways involved in BC production and fibril assembly, although the precise molecular mechanisms remain to be fully elucidated [35,37].

### 3.4. Composition of the Substrates and Wastewaters of BC Production

To verify the consumption of sugars, organic acids, and vitamins by *A. pasteurianus* ABBA during growth and BC production, the chemical composition of the OJ–SRE–GTE mixture from the test with the best performance was analyzed, along with the wastewater obtained after BC production (from both OJ and the OJ–SRE–GTE mixture). In addition, the COD was determined in the wastewater. The results are presented in Table 4.

The composition of the initial substrates SRE, OJ, and GTE, before BC production, was presented in a previous study [3]. Specifically, SRE (4 °Be) contained citric acid (0.1 g/L), tartaric acid (1.3 g/L), malic acid (1.4 g/L), glucose (4.4% *w*/*v*), and fructose (3.9% *w*/*v*), and had a TPC of 111 mg GAE/L and 2.3 mg/100 mL vitamin C. GTE contained no sugar or organic acids and exhibited a higher TPC of 179.5 mg GAE/L, providing an additional source of nitrogen and polyphenols. Also, only the OJ-SRE-GTE mixture that yielded the highest BC yield was analyzed. Compared with the SRE-OJ-GTE mixture used previously for BC production by *Komagataeibacter sucrofermentans* (50-20-30 ratio of SRE-OJ-GTE) [3], the OJ-SRE-GTE mixture (60-10-30 ratio) used in this study contained higher levels of citric acid (avg. 8.22 g/L) and malic acid (avg. 1.21 g/L), due to the higher OJ content, though still lower than in pure OJ. In contrast, the SRE-OJ-GTE mixture had lower concentrations of these acids (citric acid 3.06 g/L; malic acid 1.07 g/L). Tartaric acid in the OJ-SRE-GTE mixture (avg. 0.13 g/L) was much lower than in SRE (1.32 g/L) and the SRE-OJ-GTE mixture (0.73 g/L). Total sugars (avg. 8.42% *w*/*v*) were similar to SRE but lower than in OJ and the SRE-OJ-GTE mixture (11.0% *w*/*v*). Vitamin C (ascorbic acid) (22.0 mg/100 mL) was higher than in SRE or the SRE-OJ-GTE mixture (mean 13.2 mg/100 mL), but lower than in OJ. The TPC (365.0 mg GAE/L) exceeded the values found in SRE, OJ, GTE, and the SRE-OJ-GTE mixture (146.1 mg GAE/L) [3]. These differences arise from the different proportions of OJ, SRE, and GTE.

To assess the utilization of sugars, organic acids, ascorbic acid, and TPC by *A. pasteurianus* ABBA, the liquid residues after BC production from OJ and from the OJ-SRE-GTE mixture were analyzed. Total sugars decreased in both substrates (to 3.14 and 1.94% *w*/*v*, respectively), with sucrose showing the greatest decrease, followed by glucose, while fructose remained at relatively higher levels. This agrees with the study of Lee et al. [39], who reported the highest BC yields when sucrose was used as the C-source, followed by glucose, and then fructose.

Organic acids also decreased, with citric acid showing the largest reduction and tartaric acid the smallest in the mixture, possibly due to its inhibitory effect on BC production [27]. Ascorbic acid decreased in both substrates, indicating its utilization by this species, consistent with previous findings [3,28].

The TPC decreased to 232.0 mg GAE/L in OJ and 258.8 mg GAE/L in the mixture (a reduction of about 36% and 29%, respectively), likely due to adsorption onto BC or microbial metabolism of phenolics [8]. The volume of liquid residues after BC production was 638 mL/L of substrate for OJ and 672 mL/L for the mixture, while COD was 1.8 g/L O_2_ in both cases. In comparison, Adamopoulou et al. [3] reported 617 mL/L of wastewater with an average COD of 1.3 g/L, slightly lower than in the present work.

### 3.5. Physicochemical Characterization of BC

The SEM images (Figure S2) depict the morphology of the produced BCs by *A. pasteurianus* ABBA in both synthetic HS medium and a natural mixture of OJ-SRE-GTE; the indicated measurements, obtained during the SEM observation, are representative and illustrative. In both substrates, the BC consists of a dense network of fibrils forming a compact three-dimensional structure. The fiber diameter of the OD BC produced in the HS medium ranged from 39 to 74 nm (Figure S2a), whereas the FD sample showed fiber diameters of 35 to 82 nm (Figure S2b). For the BC produced in the OJ-SRE-GTE mixture, the fiber diameter after OD ranged from 44 to 59 nm (Figure S2c), while the FD sample exhibited fiber diameters of 41 to 78 nm (Figure S2d). The differences observed between the drying methods are likely due to the swelling that BC fibers undergo during FD [40]. Compared to the BC produced by the *A. pasteurianus* RSV-4 in HS medium, which exhibited a dense network of extremely thin fibrils with a high degree of alignment and a diameter of 42–68 nm, as shown by SEM [33], the BC in this study by *A. pasteurianus* ABBA, in both synthetic and natural substrates, displays comparable morphology and fiber diameters. Regarding the BC produced in the OJ–SRE–GTE mixture (both OD and FD), its fiber diameter is considerably smaller than that reported for BC from *K. sucrofermentans* cultivated in an SRE–OJ–GTE mixture, which exhibited diameters of 52–84 nm (OD) and 57–108 nm (FD) [3]. Finally, the fiber diameters of modified BC films from *K. sucrofermentans*, with incorporated natural zeolite) and activated carbon nanostructures and thyme oil (Zt/AC/Th), and dried at 40 °C, ranged from 42–86 nm and 39–65 nm, respectively [14].

**Figure 2:**
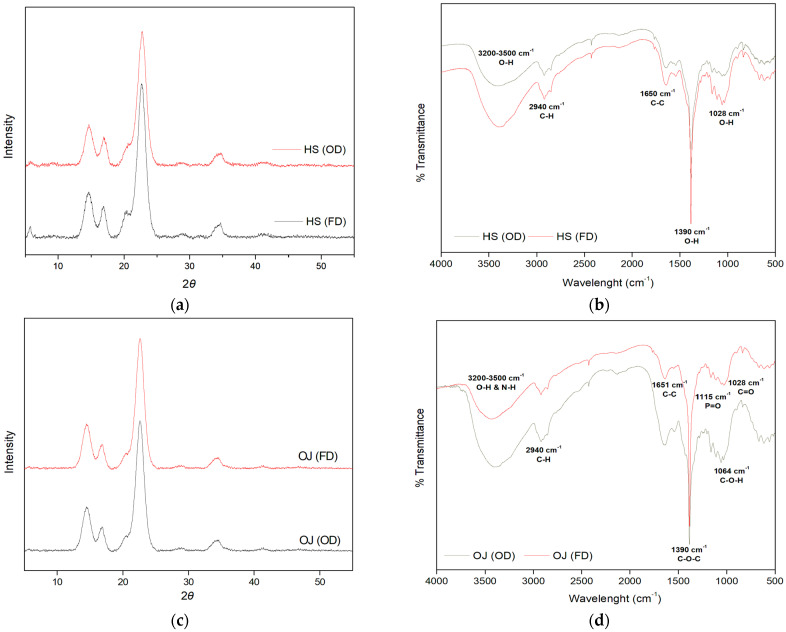

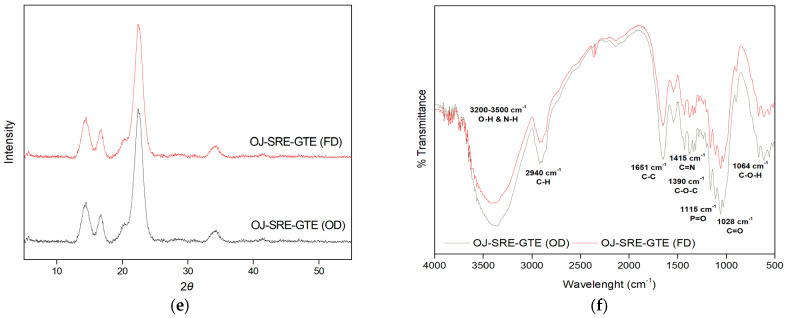
XRD spectra (**a**,**c**,**e**) and FT-IR spectra (**b**,**d**,**f**) of BC samples produced by *A. pasteurianus* ABBA. HS: Hestrin–Schramm medium. SRE: Substandard raisins extract. OJ: Orange juice. GTE: Green tea extract.

Table 5 presents the texture characteristics, and Figure 2 presents the XRD and FT-IR spectra of the BC films produced by *A. pasteurianus* ABBA. Regarding porosimetry, when comparing the two drying methods, the FD BC samples exhibit higher surface area (SA), average pore diameter (APD), and cumulative pore volume (CPV) than the OD samples. This is likely due to the swelling of the fibers during FD [40]. In relation to a previous study [6], BC produced by *K. sucrofermentans* in HS showed an SA of 6.5 m^2^/g, an APD of 201.0 Å, and a CPV of 0.043 cm^3^/g-values higher than those of OD samples produced in HS by *A. pasteurianus* ABBA. However, the FD/HS sample from *A. pasteurianus* ABBA exhibits a significantly higher SA and CPV (17.4 m^2^/g and 0.127 cm^3^/g, respectively), while its APD shows a slight increase (204.7 Å). For the samples produced in OJ and in the mixed substrate, the OJ-C (OD) sample displays higher SA, APD, and CPV than the OJ-SRE-GTE (OD). In contrast, among the FD samples, the BC from the mixture shows higher values than OJ-C (FD). Regarding FD BC, the SA and APD values (10.5 m^2^/g and 186.9 Å, respectively) are higher than those produced by *K. sucrofermentans* (5.8 m^2^/g and 180.3 Å, respectively). However, the CPV (0.088 cm^3^/g and 0.101 cm^3^/g, respectively). Conversely, in the OD samples, the SA, APD, and CPV values reported for *K. sucrofermentans* (4.8 m^2^/g, 184.7 Å, and 0.099 cm^3^/g, respectively) [3] are considerably higher than those of the BC produced by *A. pasteurianus* ABBA (Table 5).

Finally, the SA, APD, and CPV values of modified BC gels with Zt/AC/Th, produced by *K. sucrofermentans*, were considerably lower (0.7–0.8 m^2^/g, 0.006–0.01 cm^3^/g, and 165.2–203.1 Å in HS, and 0.2–0.7 m^2^/g, 0.003–0.006 cm^3^/g, and 99.3–138.4 Å in SRE, respectively). This reduction is attributed to the incorporation of the nanostructures in the BC films. In contrast, the unmodified BC produced in SRE showed an SA of 5.47 m^2^/g, a CPV of 0.084 cm^3^/g, and an APD of 264.4 Å [14].

To determine the crystalline nature of the BC produced by *A. pasteurianus* ABBA on different substrates and using two drying methods, XRD patterns were analyzed (Figure 2a,c,e). Three broad peaks appear at 2*θ* of 14.7°, 16.8°, and 22.8°, corresponding to the cellulose Iα and Iβ structures characteristic of BC. Specifically, the peak at 14.7° corresponds to the (1 0 0) and (1 1 0) crystallographic planes of cellulose Iα and Iβ, respectively; the peak at 16.8° corresponds to the (0 1 0) and (1 1 0) planes of Iα and Iβ; and the peak at 22.8° corresponds to the (1 1 0) and (2 2 0) planes of Iα and Iβ, respectively. The broadening of these characteristic peaks reflects the partial crystallinity of BC produced under static culture conditions, with the peak at 16.8° being the most characteristic of cellulose Iα synthesized by bacteria.

The XRD spectra are very similar across samples, showing only minimal differences regardless of drying method (OD, FD) or substrate used (HS, OJ, or OJ–SRE–GTE). These spectra correspond well with those previously reported for *A. pasteurianus* [33]. Furthermore, the XRD spectra of BC films produced by *K. sucrofermentans* and modified in situ with Zt/AC/Th showed no significant differences compared to the spectra described above [14]. Similarly, no differences were observed in the spectra of BC samples produced from SRE–OJ–GTE using either drying method [3]. Finally, the XRD spectra of BC produced by *K. sucrofermentans* in HS are comparable to those obtained for *A. pasteurianus* ABBA, also cultivated in HS [6].

Table 5 also presents the crystallinity index (CI) and crystallite size (CS) of the BC produced by *A. pasteurianus* ABBA. The CI is slightly higher in the OD samples or at similar levels to the FD samples, whereas the CS generally increases in the FD BC, except in the BC form OJ–SRE–GTE, where both FD and OD samples exhibit the same CS. The CI of BC produced in HS by *A. pasteurianus* MGC-N8819 and *K. sucrofermentans* strains was reported to be 70.6%, which is higher than the CI of BC produced by *A. pasteurianus* ABBA (67.1–68.3%) [6,11]. In terms of CS, the BC produced in HS by *K. sucrofermentans* (70.6–72.4 Å) [6] shows values considerably higher than those determined for BC produced in this study (66.2–69.3 Å). Comparing the CI of BC produced by *A. pasteurianus* ABBA in OJ with that of BC produced in OJ by *K. sucrofermentans*, the CI is substantially lower (65.8% vs. 86.9%, respectively) [7]. Additionally, the CI of BC from OJ–SRE–GTE in this study (63%) is similar to that of BC from SRE–OJ–GTE (OD) produced by *K. sucrofermentans* (63.2%) but significantly lower than that of the corresponding FD BC (74.7%). The CS of BC from OJ–SRE–GTE in this study (69 Å) is notably higher than that of BC from SRE–OJ–GTE (OD) (60.2 Å), yet considerably lower than that of BC from SRE–OJ–GTE (FD) (74.2 Å) produced by *K. sucrofermentans* [3].

The reduced crystallinity observed in BC produced by *A. pasteurianus* ABBA cultivated in OJ or in OJ–SRE–GTE, compared to that produced in HS, is likely due to the presence of ascorbic acid, which can form H-bonds with BC, thereby reducing its crystallinity [28]. Finally, the CI and CS values of the OD samples are similar to or slightly lower than those reported for BC gels by *K. sucrofermentans* and modified with Zt/AC/Th, where CI ranged from 65–69.6% and CS from 71.7–73.8 Å in both HS and SRE substrates [14].

Figure 2b presents the FT-IR spectra of BC produced in HS by *A. pasteurianus* ABBA. Peaks were detected at 3200–3500, 2940, 1650, 1390, and 1028 cm^−1^, corresponding to the vibrations of O–H, C–H, C–C, C–O–C, and C=O bonds, respectively. Similar peaks have been reported for BC produced by *A. pasteurianus* in HS [33,34]. Figure 2d,f present the FT-IR spectra of BC produced on OJ and OJ–SRE–GTE, respectively. In addition to the characteristic peaks observed for the BC/HS sample, the absorption band at 3200–3500 cm^−1^ corresponds to O–H and N–H groups, likely attributed to the presence of GTE and H-bonding, and appears broad in almost all samples [3,6,35]. The peak at 1650 cm^−1^ is associated with C–C bonds, possibly indicating the presence of ascorbic acid on the BC film. Additionally, the peaks at 1433 and 1128 cm^−1^ correspond to C=N and P=O bonds, respectively, likely due to thiamine present on the BC film [3,36,41].

Regarding the antioxidant activity (Table 5) of BC from OJ–SRE–GTE, it is higher than that of BC from OJ-C. This increase is likely due to the presence of ascorbic acid on the BC, as suggested by the FT-IR spectra (Figure 2d,f), as well as due to the adsorption of phenolic compounds from the GTE [8,36]. Furthermore, the FD samples exhibit higher antioxidant activity compared to the OD samples. This is likely attributed to fiber swelling and the formation of larger pores in FD samples, resulting in more accessible sites for the incorporation of bioactive compounds through H-bonding within the BC matrix [8,28,40,42]. The antioxidant activity of BC from OJ–SRE–GTE (OD) (43.22%) is slightly lower than that of BC from SRE–OJ–GTE (OD) produced by *K. sucrofermentans* (47.22%), while the antioxidant activity of BC from OJ–SRE–GTE (FD) (50.61%) is slightly higher compared to BC from SRE–OJ–GTE (FD) (49.38%) [3]. These differences are likely explained by the varying proportions of OJ, SRE, and GTE in the natural substrates used for BC production in these studies, as well as the structural differences in BC produced by the two different bacteria, *A. pasteurianus* ABBA and *K. sucrofermentans*.

Finally, the FT-IR spectra of the BC films produced in the natural substrates exhibited characteristic peaks corresponding to gallic acid, ascorbic acid, and thiamine, indicating their adsorption onto the BC matrix and contributing to the enhanced antioxidant activity. A direct comparison with BC produced in an artificial medium supplemented with pure standards of these compounds would further strengthen this conclusion. However, the absence of these characteristic peaks in the HS-derived BC films provides strong qualitative evidence for the adsorption of these bioactive compounds and supports the proposed interpretations.

## 4. Conclusions

The new BC-producing AAB strain isolated from spontaneous wine fermentations was identified as *A. pasteurianus*. The ability of this strain to synthesize BC on both synthetic and natural media demonstrates its metabolic versatility and relevance for sustainable bioprocess development. Comprehensive structural and physicochemical characterization showed that BC properties are influenced by both the cultivation substrate and the post-treatment (drying) method, affecting features such as porosity, crystallinity, and antioxidant-related functionality. The study further demonstrates that natural substrates derived from agrifood sidestreams, including oranges and raisin processing sidestreams, and tea extracts as additional nitrogen and polyphenol sources, can effectively support BC biosynthesis. Process optimization using RSM enabled the identification of suitable substrate combinations and cultivation conditions, reinforcing the value of statistical tools for developing efficient and robust BC production systems based on complex raw materials. Overall, the co-utilization of regionally abundant agro-industrial residues for BC production highlights a sustainable and circular-economy-oriented approach for generating value-added biomaterials. This work provides a foundation for future studies aimed at process scale-up, dynamic cultivation strategies, and expanded applications of BC produced from renewable resources.

## Figures and Tables

**Table 1:** Microbiological characteristics of the isolated strain.

Microbial Analysis	New Strain
Morphology	Rod
Gram strain	Gram-negative
Catalase	Positive
Oxidase	Negative
Acetic acid production	Positive
Acetic acid peroxidation	Positive
Bacterial cellulose production	Positive
Ethanol oxidation	Positive
Production of dihydroxyacetone	Negative

**Table 2:** Concentration (ng/μL) and purity (A_260_/A_280_) of the isolated DNA of the new strain.

Strain	DNA Concentration (ng/L)	A_260_/A_280_
New strain	44.5	1.88

**Table 3:** Experimental RSM/CCD design for the optimization of BC production, under static conditions in OJ, and experimental and predicted values of the BC yields.

Test	Independent Variables (Substrate Concentration)				Dependent Variable	
	Coded Values		Actual Values		BC Yield (g/L)	
	Χ_1_ (SRE)	Χ_2_ (GTE)	SRE (%*v*/*v*)	GTE (%*v*/*v*)	Experimental Value	Predicted Value
1	−1	−1	0	0	5.01 ± 0.05	6.34
2	1	−1	20	0	8.91 ± 0.03	9.25
3	−1	1	0	60	2.82 ± 0.01	2.32
4	1	1	20	60	1.08 ± 0.00	0.41
5	−1	0	0	30	8.47 ± 0.04	7.64
6	1	0	20	30	6.58 ± 0.02	7.73
7	0	−1	10	0	14.5 ± 0.16	12.73
8	0	1	10	60	3.99 ± 0.04	5.98
9	0	0	10	30	14.81 ± 0.03	12.82
10	0	0	10	30	15.44 ± 0.02	12.82
11	0	0	10	30	13.88 ± 0.03	12.82
12	0	0	10	30	15.20 ± 0.03	12.82
13	0	0	10	30	14.57 ± 0.01	12.82

BC: Bacterial cellulose. OJ: Orange juice SRE: Substandard raisins extract (4 °Be). GTE: Green tea extract.

**Table 4:** Chemical composition of the substrates and wastewaters after BC production (in the optimum substrates).

Parameter	Substrate	Wastewater	
Sugars (%*w*/*w*)	OJ-SRE-GTE	OJ	OJ-SRE-GTE
Total	8.42 ± 0.05 ^c^	3.14 ± 0.03 ^d^	1.94 ± 0.02 ^e^
Glucose	3.09 ± 0.04 ^b^	1.04 ± 0.03 ^c^	0.81 ± 0.01 ^d^
Fructose	2.62 ± 0.02 ^b^	1.12 ± 0.03 ^c^	0.92 ± 0.01 ^d^
Sucrose	2.72 ± 0.02 ^b^	0.97 ± 0.03 ^c^	0.22 ± 0.01 ^d^
Organic acids (g/L)			
Citric acid	8.22 ± 0.01 ^c^	7.23 ± 0.05 ^d^	5.09 ± 0.06 ^e^
Tartaric acid	0.13 ± 0.02 ^b^	nf	0.11 ± 0.02 ^b^
Mallic acid	1.21 ± 0.02 ^b^	1.12 ± 0.02 ^c^	1.03 ± 0.03 ^d^
Vitamin C (mg/100 mL)	22.0 ± 0.4 ^c^	22.3 ± 0.9 ^c^	12.8 ± 0.04 ^d^
TPC (mg GAE/L)	365.0 ± 0.7 ^d^	232.0 ± 1.1 ^e^	258.8 ± 0.7 ^f^
COD (g/L)		1.8 ± 0.0 ^a^	1.8 ± 0.0 ^b^

SRE: Substandard raisins extract. OJ: Orange juice. GTE: Green tea extract. nf: not found. Superscript letters in a row indicate statistical differences between treatments (*p* < 0.05) according to One-Way ANOVA analysis.

**Table 5:** Textural characteristics and antioxidant activity of the BC films by *A. pasteurianus* ABBA.

Parameter	Substrate					
	HS		OJ-C		OJ-SRE-GTE	
	OD	FD	OD	FD	OD	FD
SA (m^2^/g)	3.9	17.4	4.0	4.8	3.4	10.5
APD (Å)	135.2	204.7	182.5	182.5	148.8	186.9
CPV (cm^3^/g)	0.030	0.127	0.032	0.038	0.066	0.088
CI (%)	68.3	67.1	65.8	64.9	63.0	63.0
CS (Å)	66.2	69.3	66.0	70.0	69.0	69.0
AA (%)			21.70 ± 0.09 ^a^	30.07 ± 0.07 ^b^	43.22 ± 0.25 ^c^	50.61 ± 0.25 ^d^

HS: Hestrin–Schramm medium. SRE: Substandard raisins extract. OJ: Orange juice. GTE: Green tea extract. C: Control. SA: Surface area. APD: Average pore diameter. CPV: Cumulative pore volume. CI: Crystallinity index. CS: Crystallite size: AA: Antioxidant activity. Superscript letters in a row indicate statistical differences between treatments (*p* < 0.05) according to One-Way ANOVA analysis.

## Data Availability

The original contributions presented in this study are included in the article/Supplementary Material. Further inquiries can be directed to the corresponding author.

## Supplementary Materials

The following supporting information can be downloaded at: https://www.mdpi.com/article/10.3390/foods15010154/s1. Figure S1. Electrophoresis results: (a) New strain (*A. pasteurianus* ABBA). (b) Base pair range of the DNA molecular marker (M). -ve: negative sample (IT and NL primers). 4,5,6: New strain sample with the 16Sd-16Sr primers, respectively. Figure S2. SEM images of dried BC films by *A. pasteurianus* ABBA produced in HS medium, (a) OD (×3000), and (b) FD (×3000), and in a mixture of OJ-SRE-GTE, (c) OD (×2500), and (d) FD (×2500). Table S1. Independent variables, their coded and actual values, for the RSM/CCD optimization of BC production, under static conditions, in OJ. Table S2. Results of sequencing with the Sanger method. Table S3. ANOVA of the RSM/CCD model for optimizing BC production in a mixture of orange juice with independent variables: raisin extract and green tea extract.

## Abbreviations

The following abbreviations are used in this manuscript:

BC	Bacterial Cellulose
SRE	Substandard Raisin Extracts
OJ	Orange Juice
GTE	Green Tea Extract
HS	Hestrin–Schramm Medium
OD	Oven-Dried
FD	Freeze-Dried
AAB	Acetic Acid Bacteria
RSM	Response Surface Methodology
CCD	Central Composite Design
GY	Glucose–Yeast Extract Medium
GYC	GY–CaCO_3_ Medium
EYC	Ethanol–Yeast Extract–CaCO_3_ Medium
DHA	Dihydroxyacetone
PCR	Polymerase Chain Reaction
AGE	Agarose Gel Electrophoresis
EDTA	Ethylenediaminetetraacetic acid
TAE	Tris-Acetate-EDTA
Etbr	Ethidium Bromide
TPC	Total Phenolic Content
XRD	X-Ray Diffraction
FT-IR	Fourier-Transform Infrared Spectroscopy
SEM	Scanning Electron Microscopy
COD	Chemical Oxygen Demand
SA	Surface Area
APD	Average Pore Diameter
CPV	Cumulative Pore Volume
CI	Crystallinity Index
CS	Crystallite Size
