# Supplementary material for: Isolation of a New Acetobacter pasteurianus Strain from Spontaneous Wine Fermentations and Evaluation of Its Bacterial Cellulose Production Capacity on Natural Agrifood Sidestreams
Source: Foods. 2026 Jan 3;15(1):154. doi: 10.3390/foods15010154 (PMC12786161; doi:10.3390/foods15010154)
Supplement: Supplementary file 1 [file foods-15-00154-s001.zip › foods-4048959-supplementary.pdf]

Article

Isolation of a new *Acetobacter pasteurianus* strain from spontaneous wine fermentations and evaluation of its bacterial cellulose production capacity on natural agrifood sidestreams

Vasiliki Adamopoulou, Vasiliki Karakovouni, Panagiota Michalopoulou, Panagiota M. Kalligosfyri, Agapi Dima, Theano Petsi, Despina P. Kalogianni, and Argyro Bekatorou*

Department of Chemistry, University of Patras, Patras, 26504, Greece

Supplementary material

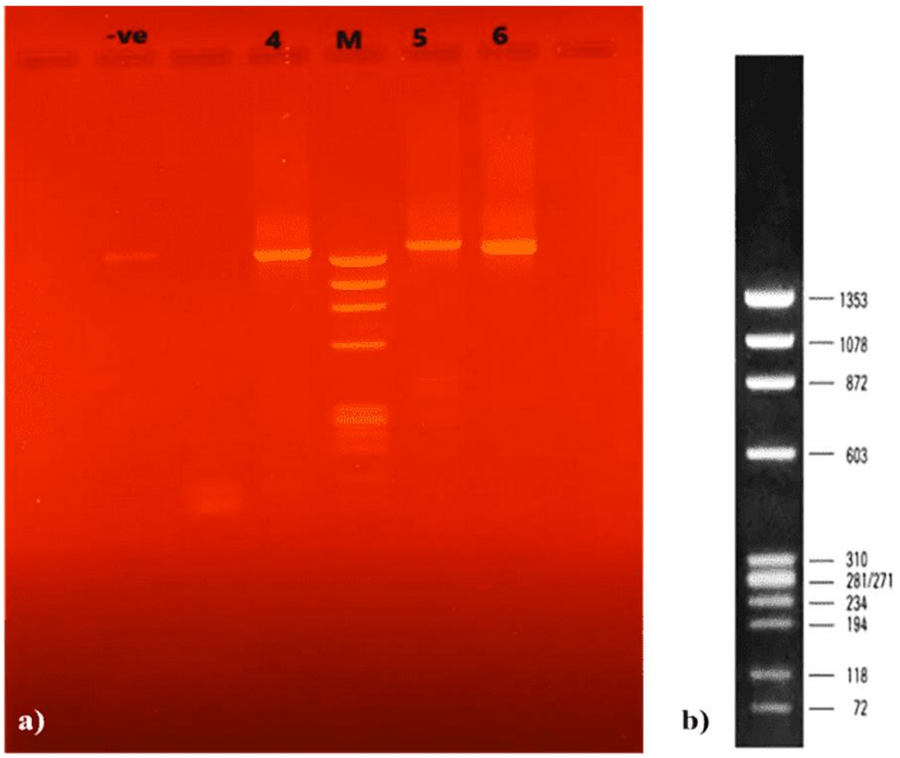

Figure S1. Electrophoresis results: a) New strain (*A. pasteurianus* ABBA). b) Base pair range of the DNA molecular marker (M). -ve: negative sample (IT and NL primers). 4,5,6: New strain sample with the 16Sd-16Sr primers, respectively.

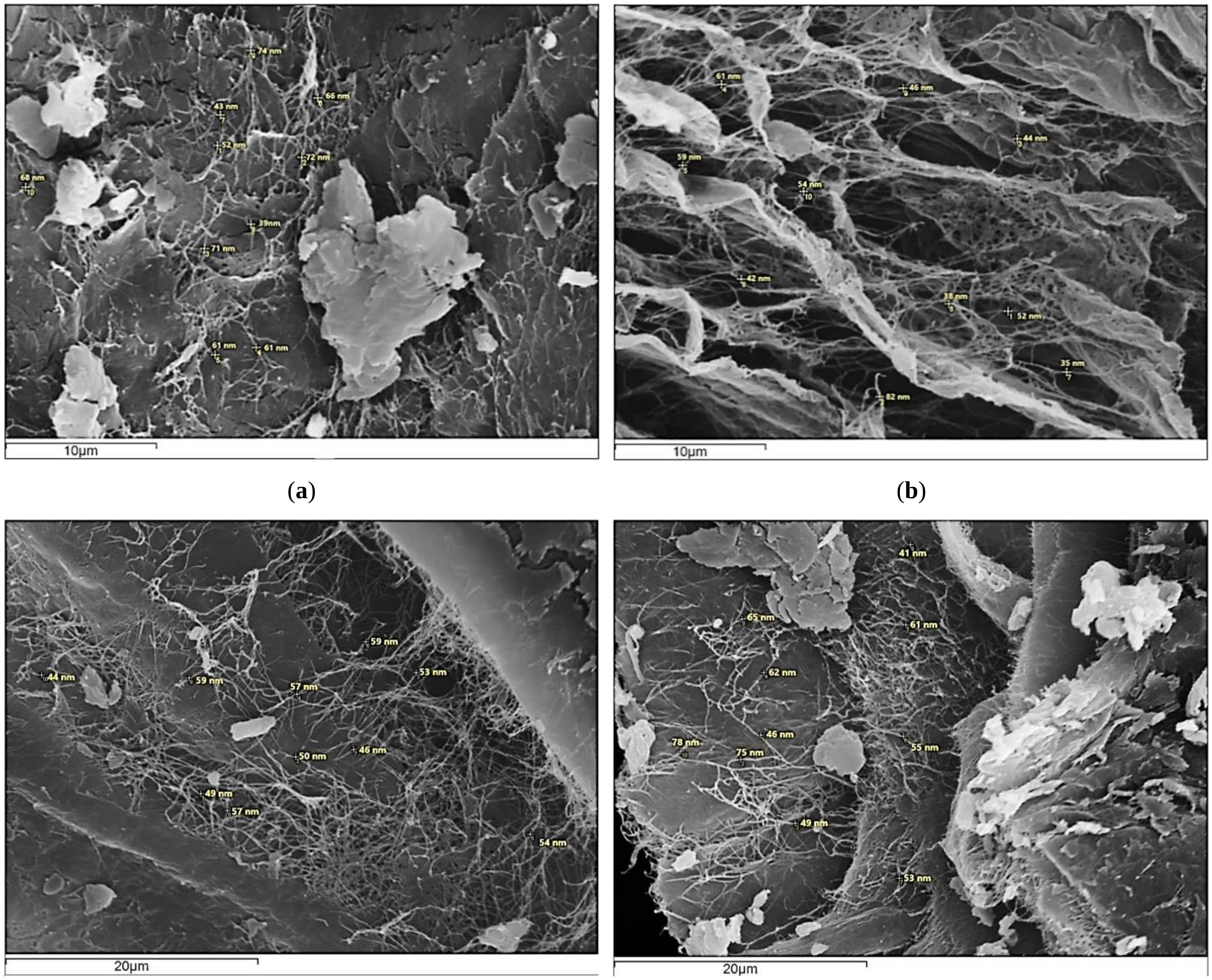

(**a**) (**b**) (**c**) (**d**)

Figure S2. SEM images of dried BC films by *A. pasteurianus* ABBA produced in HS medium, (a) OD (×3000), and (b) FD (×3000), and in a mixture of OJ-SRE-GTE, (c) OD (×2500) and (d) FD (×2500).

Table S1. Independent variables, their coded and actual values, for the RSM/CCD optimization of BC production, under static conditions, in OJ.

Independent variable		Symbol	Coded values		
			-1	0	1
SRE	%v/v	X_1	0.00	10.00	20.00
GTE	%v/v	X_2	0.00	30.00	60.00

SRE: Substandard raisins extract. GTE: Green tea extract. OJ: Orange juice. RSM/CCD: Response Surface Methodology/Central Composite Design.

Table S2. Results of sequencing with the Sanger method.

>20220609DS2P2_F05_2022-06-15.ab1

GCGGACSACKCTAGTCGGCTGCGTCCTTGMGGTTCGCTMCCGRCTTARGGTSWACCAACTCCCATGGTGTGACGGGCGGT
GTGTACAAGGCCCGGGAACGTATTCACCGCGGCATGCTGATCCSCGATTACTARCGATTCCACCTTCMTGCACTCSAATT
GCARASTGMAATCCSAACTGARACRGCTTTTTRAGATAAKCATGGTGTCWCCACCTATCTTCCCACTGTCACCGCCMTTG
TAKCACGTGTGTAKCCCASGAGATAARGGGCATGAGGACATGACATCWTCCCCACCTTCCTCCGGCTTGTCWCCGGSRST
CTCTCWASAGAGYCCMCMCCWWMMTGATGGCAACTAAWRATARGGGTTGCGCTCGTTGCGGGACTTAACCCAACATCTCA
CGACACGAGCTGACGACARCCATGCASCACCTGTGTYMRAGGTCCCTTGCGGAAAATMWACATCTCTGCATGCGSCCTCT
ACGTTCAAACCCGGGWAAGGTTTTGCCGKATGYTTCAAATTAAACCCAWGGCCCCACCCKTTGGGGGGGCCCCCSYMAAT
TCCTTTRAWTTTMAACCTTGGGGCCKWAWCCCCCAGGGGGTGGGGTTAAGCGTTTKCTGASAACTGAAAGGARKAAACMC
CCAACATCTYAGCAYTCATCGTTTACGGYRTGGACTACCAGGTATCTAATCCTGTTTGCTMCCCATACTTTCGMGCCTCA
GCGTCAGTWAYGAGACCAGAYWGCCGCCTTCGCCACTGGTGTTCTTCCAATATCTACGMATTTCACCKCTACACTKGGAG
TTCCACWGYCTCTTCASMCTCTAGTCTGCAMGTWTCAGATGCACTCCTMGGTTRAGCCGRGGATTTCACATCWGACTGTA
CAAACGCTRMGCKCCCTTTACGCCAGTCAATCGAGCACGCTTGCCCCTACGTATACGCGGCTGCTGGCACGWAGTAGCGK
GCTTCTTYKRYGRTACCGTCATCATCCKKMCAGYGAACGTGCATACGATCTGATACCTCCTMCTCTACGACCTGAYTSAT
CATGCTGGCGCGATGTSCATATCCAMTGCKCATCGGTAGATYCGACKGTCATCAGWGTYGACKGCYGACAGWGCATGCTG
AGCTACTACATACGTTACGTAGTCCAAGCATGTCTGACCCTAGGTCWGCSAWGC

R: A or G. Y: C, T or U. K: G, T or U. M: A or C. S: C or G. W: A, T or U

Table S3. ANOVA of the RSM/CCD model for optimizing BC production in a mixture of orange juice with independent variables: raisins extract and green tea extract.

Source	Sum of Squares	Degrees of freedom	Mean square	F-Value	Probability (P)>F
Model	236.37	5	47.27	3.35	0.0073
Residual	8.84	7	0.42		
Lack of fit	3.17	3	1.39	0.89	0.0205
Pure error	1.67	4	0.42		
Total	250.05	12			

R^2=0.71, CV=4.38%, $RAdj^2$=0.89, Pred R^2=0.72
